# Supplementary material for: Effects of intestinal parasite infection on hematological profiles of pregnant women attending antenatal care at Debre Markos Referral Hospital, Northwest Ethiopia: Institution based prospective cohort study
Source: PLoS One. 2021 May 10;16(5):e0250990. doi: 10.1371/journal.pone.0250990 (PMC8109803; doi:10.1371/journal.pone.0250990)
Supplement: Attachment — Submitted filename: Review of the manuscript ID PONE-D-20-36685.pdf [file pone.0250990.s004.pdf]

PLOS ONE

Effects of intestinal parasite infection on hematological profile of Pregnant Women Attending Antenatal care in Debre Markos Referral Hospital, North West Ethiopia. Institution ba[illegible]ed Prospective cohort study

--Manuscript Draft--

**Manuscript Number:**	PONE-D-20-36685
**Article Type:**	Research Article
**Full Title:**	Effects of intestinal parasite infection on hematological profile of Pregnant Women Attending Antenatal care in Debre Markos Referral Hospital, North West Ethiopia. Institution based Prospective cohort study
**Short Title:**	Effects of intestinal parasite infection on hematological profile of Pregnant Women
**Corresponding Author:**	Gebreselassie Demeke, MSc<br>Debre Markos University<br>Debre Markos, ETHIOPIA
**Keywords:**	Pregnant women, intestinal parasites, anemia, hematological profile, concentration method
**Abstract:**	Back ground : Intestinal parasitosis is common diseases that cause misery and disability in poor populations. The numbers of individuals affected are staggering. From two billion peoples who harbor parasites worldwide, 300 million suffer severe morbidity. More than 25% of pregnant women are infected with hookworm, which causes intestinal bleeding and blood loss, and has been most commonly associated with anemia. Parasite infection in pregnancy have been associated with iron deficiency, maternal anemia, and impaired nutritional status, as well as decreased infant birth weight.<br><br>Objective : to assess the effects of intestinal parasite infection on hematological profile of Preg[illegible] Women Attending Antenatal care in Debre Markos Referral Hospital from December 2017 to February 2019.<br><br>Method : A Cohort study design was conducted on pregnant women who followed anten[illegible]care at Debre Markos Referral hospital from December/ 2017 to February 2019. The study was conducted by taking blood, stool and mid upper arm circumference measurement in every trimester Data was analyzed using SPSS version 24 software. Data was presented using tables. Association of hemoglobin levels with intestinal parasitic infection was analyzed using logistic regression analyses and the mean hematological profile difference between parasite infected and not infected pregnant women was analyzed using independent t-test.<br><br>Result . In the present study the predominant parasite were Enteamoeba histolytica , hoo[illegible]rm, Giardia lamblia Schistosoma mansoni and Ascaris lumbricoid. The mean HGB, HCT MCV, MCH and MCHC of intestinal parasite infected pregnant women were 12.8g/dl, 38.2%, 94.7fl, 33.1pg and 34.7g/dl respectively. But pregnant women who were free from intestinal parasite their HGB, HCT, MCV, MCH and MCHC were 14.4 g/dl, 39.8%, 94.9fl, 33.9pg and 35.5g/dl respectively. Anemia was strongly associated with hookworm (AOR=21.29, 95%CI: 8.28-54.75, P<0.001), S.mansoni (AOR=63.73, 95% CI: 19.15-212, p<0.001) and A.lumbricoid (AOR=14.12, 95% CI 3.28-60.65, P<0.001).<br><br>Conclusion . Intestinal parasite infection in pregnant women had adverse impact on hematol[illegible]al profile. Which leads anemia in pregnant women as well as on infant and also under birth weight. As to minimize maternal anemia and under birth weight deworming is better before pregnancy.
**Order of Authors:**	Gebreselassie Demeke, MSc
	Getachew Mengistu
	Abtie Abebaw

Powered by Editorial Manager® and ProduXion Manager® from Aries Systems Corporation

	Milkiyas Toru
	Molla Yigzaw
	Aster Shiferaw
	Hylemariam Mihiretie Mengist
	Tebelay Dilnessa
**Additional Information:**	
**Question**	**Response**
**Financial Disclosure**<br><br>Enter a financial disclosure statement that describes the sources of funding for the work included in this submission. Review the submission guidelines for detailed requirements. View published research articles from *PLOS ONE* for specific examples.<br><br>This statement is required for submission and **will appear in the published article** if the submission is accepted. Please make sure it is accurate.<br><br>**Unfunded studies**<br>Enter: *The author(s) received no specific funding for this work.*<br><br>**Funded studies**<br>Enter a statement with the following details:<br>• Initials of the authors who received each award<br>• Grant numbers awarded to each author<br>• The full name of each funder<br>• URL of each funder website<br>• Did the sponsors or funders play any role in the study design, data collection and analysis, decision to publish, or preparation of the manuscript?<br>• **NO** - Include this sentence at the end of your statement: *The funders had no role in study design, data collection and analysis, decision to publish, or preparation of the manuscript.*<br>• **YES** - Specify the role(s) played.<br><br>* typeset	Fund received author: Gebreselassie Demeke<br>Funder :-Debre Debre Markos University<br><br>The funders had no role in study design, data collection and analysis, decision to publish, or preparation of the manuscript.
**Competing Interests**<br><br>Use the instructions below to enter a competing interest statement for this submission. On behalf of all authors,	The authors have declared that no competing interests exist.

disclose any competing interests that could be perceived to bias this work—acknowledging all financial support and any other relevant financial or non-financial competing interests.<br><br>This statement **will appear in the published article** if the submission is accepted. Please make sure it is accurate. View published research articles from *PLOS ONE* for specific examples.<br><br>**NO authors have competing interests**<br><br>Enter: *The authors have declared that no competing interests exist.*<br><br>**Authors with competing interests**<br><br>Enter competing interest details beginning with this statement:<br><br>*I have read the journal's policy and the authors of this manuscript have the following competing interests: [insert competing interests here]*<br><br>* typeset	
**Ethics Statement**<br><br>Enter an ethics statement for this submission. This statement is required if the study involved:<br><br>• Human participants<br>• Human specimens or tissue<br>• Vertebrate animals or cephalopods<br>• Vertebrate embryos or tissues<br>• Field research<br><br>Write "N/A" if the submission does not require an ethics statement.<br><br>General guidance is provided below. Consult the submission guidelines for detailed instructions. **Make sure that all information entered here is included in the Methods section of the manuscript.**	Ethical clearance was obtained from the research ethical committee of College of Health Science,Debre Markos University. All the information obtained from the study subjects was coded to keep confidential. The study participants were signed written consent.

Powered by Editorial Manager® and ProduXion Manager® from Aries Systems Corporation

**Format for specific study types**<br><br>**Human Subject Research (involving human participants and/or tissue)**<br>• Give the name of the institutional review board or ethics committee that approved the study<br>• Include the approval number and/or a statement indicating approval of this research<br>• Indicate the form of consent obtained (written/oral) or the reason that consent was not obtained (e.g. the data were analyzed anonymously)<br><br>**Animal Research (involving vertebrate animals, embryos or tissues)**<br>• Provide the name of the Institutional Animal Care and Use Committee (IACUC) or other relevant ethics board that reviewed the study protocol, and indicate whether they approved this research or granted a formal waiver of ethical approval<br>• Include an approval number if one was obtained<br>• If the study involved *non-human primates*, add *additional details* about animal welfare and steps taken to ameliorate suffering<br>• If anesthesia, euthanasia, or any kind of animal sacrifice is part of the study, include briefly which substances and/or methods were applied<br><br>**Field Research**<br><br>Include the following details if this study involves the collection of plant, animal, or other materials from a natural setting:<br>• Field permit number<br>• Name of the institution or relevant body that granted permission	
**Data Availability**<br><br>Authors are required to make all data underlying the findings described fully available, without restriction, and from the time of publication. PLOS allows rare exceptions to address legal and ethical concerns. See the PLOS Data Policy and FAQ for detailed information.	Yes - all data are fully available without restriction

Powered by Editorial Manager® and ProduXion Manager® from Aries Systems Corporation

A Data Availability Statement describing where the data can be found is required at submission. Your answers to this question constitute the Data Availability Statement and **will be published in the article**, if accepted.<br><br>**Important:** Stating 'data available on request from the author' is not sufficient. If your data are only available upon request, select 'No' for the first question and explain your exceptional situation in the text box.<br><br>Do the authors confirm that all data underlying the findings described in their manuscript are fully available without restriction?	
**Describe where the data may be found in full sentences. If you are copying our sample text, replace any instances of XXX with the appropriate details.**<br><br>• If the data are **held or will be held in a public repository**, include URLs, accession numbers or DOIs. If this information will only be available after acceptance, indicate this by ticking the box below. For example: *All XXX files are available from the XXX database (accession number(s) XXX, XXX.*).<br>• If the data are all contained **within the manuscript and/or Supporting Information files**, enter the following: *All relevant data are within the manuscript and its Supporting Information files.*<br>• If neither of these applies but you are able to provide **details of access elsewhere**, with or without limitations, please do so. For example:<br><br>*Data cannot be shared publicly because of [XXX]. Data are available from the XXX Institutional Data Access / Ethics Committee (contact via XXX) for researchers who meet the criteria for access to confidential data.*<br><br>*The data underlying the results presented in the study are available*	All relevant data are within the manuscript and its Supporting Information files

from (include the name of the third party and contact information or URL).<br>• This text is appropriate if the data are owned by a third party and authors do not have permission to share the data.<br><br>* typeset	
Additional data availability information:	

Manuscript Click here to access/download;Manuscript;manuscript.docx

Effects of intestinal parasite infection on hematological profile of Pregnant Women Attending Antenatal care in Debre Markos Referral Hospital, North West Ethiopia. Institution based Prospective cohort study

Gebreselassie Demeke [1*], Getachew Mengistu [2], Abtie Abebaw[3], Milkiyas Toru [4], Molla Yigzaw [5], Aster Shiferaw[6], Hylemariam Mihiretie[7] Tebelay Dilnessa (MSc.) [8]

1. Department of Medical Laboratory Science, College Health Science, Debre Markos University, Debre Markos, Ethiopia; e-mail: gebredemeke@yahoo.com
2. Department of Medical Laboratory Science, College Health Science, Debre Markos University, Debre Markos, Ethiopia; e-mail: gechomlt@gmail.com
3. Department of Medical Laboratory Science, College Health Science, Debre Markos University, Debre Markos, Ethiopia; e-mail: aabtie@gmail.com
4. Department of Medical Laboratory Science, College Health Science, Debre Markos University, Debre Markos, Ethiopia; e-mail: milkiastoru@gmail.com
5. Department of Medical Laboratory Science, College Health Science, Debre Markos University, Debre Markos, Ethiopia; e-mail: mollayigzaw33@gmail.com
6. Department of Midwifery, College Health Science, Debre Markos University, Debre Markos, Ethiopia; e-mail: astershiferaw21@gmail.com
7. Department of Medical Laboratory Science, College Health Science, Debre Markos University, Debre Markos, Ethiopia; e-mail: hylemariam@gmail.com
8. Department of Medical Laboratory Science, College Health Science, Debre Markos University, Debre Markos, Ethiopia; e-mail: tebelay@gmail.com

[*]Corresponding author

E-mail: gebredemeke@yahoo.com

Abstract:

Back ground: Intestinal parasitosis is common diseases that cause misery and disability in poor populations. The numbers of individuals affected are staggering. From two billion peoples who harbor parasites worldwide, 300 million suffer severe morbidity. More than 25% of pregnant women are infected with hookworm, which causes intestinal bleeding and blood loss, and has been most commonly associated with anemia. Parasite infection in pregnancy have been associated with iron deficiency, maternal anemia, and impaired nutritional status, as well as decreased infant birth weight.

Objective: to assess the effects of intestinal parasite infection on hematological profile of Pregnant Women Attending Antenatal care in Debre Markos Referral Hospital from December 2017 to February 2019.

Method: A Cohort study design was conducted on pregnant women who followed antenatal care at Debre Markos Referral hospital from December/ 2017 to February 2019. The study was conducted by taking blood, stool and mid upper arm circumference measurement in every trimester Data was analyzed using SPSS version 24 software. Data was presented using tables. Association of hemoglobin levels with intestinal parasitic infection was analyzed using logistic regression analyses and the mean hematological profile difference between parasite infected and not infected pregnant women was analyzed using independent t-test.

Result. In the present study the predominant parasite were *Enteamoeba histolytica*, hookworm, *Giardia lamblia Schistosoma mansoni* and *Ascaris lumbricoid.* The mean HGB, HCT MCV, MCH and MCHC of intestinal parasite infected pregnant women were 12.8g/dl, 38.2%, 94.7fl, 33.1pg and 34.7g/dl respectively. But pregnant women who were free from intestinal parasite their HGB, HCT, MCV, MCH and MCHC were 14.4 g/dl, 39.8%, 94.9fl, 33.9pg and 35.5g/dl respectively.

Anemia was strongly associated with hookworm (AOR=21.29, 95%CI: 8.28-54.75, $P<0.001$), *S.mansoni* (AOR=63.73, 95% CI: 19.15-212, $p<0.001$) and *A.lumbricoid* (AOR=14.12, 95% CI 3.28-60.65, $P<0.001$).

Conclusion. Intestinal parasite infection in pregnant women had adverse impact on hematological profile. Which leads anemia in pregnant women as well as on infant and also under birth weight. As to minimize maternal anemia and under birth weight deworming is better before pregnancy.

Key words: Pregnant women, intestinal parasites, anemia, hematological profile, concentration method

Introduction

Intestinal parasites are live in gastro intestinal tracts of human. From these, Soil-transmitted helminths (STHs) inflict a substantial morbidity burden on poor populations living in tropical and subtropical regions[1].

Intestinal parasitosis is common diseases that continue to cause misery and disability in poor populations. The numbers of individuals affected are staggering. From two billion peoples who harbor parasites worldwide, 300 million suffer severe morbidity. In 1999, World Health Organization (WHO) estimated that schistosomiasis and soil-transmitted helminthiasis represented more than 40% of the disease burden due to all tropical diseases, excluding malaria [2].
In the developing world, pregnant women, and their infants and children frequently experience a under nutrition (macronutrient and micronutrient) and repeated infection, including parasitic infections, lead to adverse consequences that can continue from one generation to the next.[3]
Tropical diseases such as intestinal helminthes, filariasis, and malaria have high impact on reproductive health. Many cases of unexplained pregnancy loss are due to undiagnosed tropical diseases. Malnutrition or anemia caused by intestinal worms may be worsened by pregnancy and make the pregnancy difficult [3].

Although anemia in pregnancy is multi-factorial, poor nutrition and infection are common causes. In Sub-Saharan Africa, soil transmitted helminthes (STH) including hookworm, urogenital schistosomiasis, and other parasitic infections such as malaria contribute to the high anemia rates in women and young children [4].

Helminthic infection prevalence up to 50% has been documented in some regions in Sub-Saharan Africa [5]. More than 25% of pregnant women are infected with hookworm, which causes intestinal bleeding and blood loss, and has been most commonly associated with anemia [5, 6].

Method and Materials

Study area, design and period

Prospective cohort study was conducted among pregnant women attending antenatal care at Debre Markos Referral Hospital, Ethiopia from December/ 2017 to February 2019.

Study population and sample size determination

Pregnant women attending antenatal care and who fulfil inclusion criteria were considered as Study population. The sample size was calculated by EpiInfo version 7.2.1.0 software (CDC, USA) using the unexposed to exposed ratio with 5% level of significant and 80% power; and by considering prevalence of anemia 10% and 21.7% among helminthic non infected and helminthic infected pregnant women, respectively. Pregnant women who attend antenatal care (ANC) unit at Debre Markos referral hospital during the study period.

Exclusion criteria

Those have known chronic disease and HIV and have taken anti-helminthic drugs within the last two weeks.

Data collection instrument and laboratory procedure

Before starting data collection, each participant was informed about the objective of the study. The study participants were signed written consent. Socio demographic data was collected using questionnaire, laboratory data and other clinical data was collected at first, second and third trimester.

Stool specimen was collected from each participant using leak proof sterile stool cup. Freshly void stool specimen was directly examined microscopically to identify intestinal parasite trophoizite. The rest 1gm stool was preserved with 10% formalin for further concentration method.

Formol-Ether concentration method

Stool specimen was processed following formol-ether standard operation procedure.1gm of stool was in a clean conical centrifuge tube containing 7ml ,of 10 % formol water. The suspension was filtered through a sieve in to 15ml conical centrifuge tube. Then 4 ml of diethyl ether was added to the formalin solution, the content was centrifuged at 300rpm for 1minute.The supernatant was discarded smear prepared using slide from the sediment. Finally the slide was examined under microscope with magnification power of 10x objective lenses[7].

Complete blood cell counts

2ml of venous blood was collected using syringe with needle and transfer to EDTA tube for hematological analysis. Complete blood cell count was analyzed using hematological analyze (mind ray BC-3000 plus).

Pregnant women hemoglobin level equal to or greater than 11g/dl considered as normal and, 10-10.9g/dl is mild, 7-7.99g/dl moderate, < 7g/dl considered as severe anemia[8]. In addition to biological sample, anthropometric measurement was measured to assess nutritional status.

Statistical Analysis

Data was double entered to EPI Data and analyzed using SPSS version 24 software. Data was presented using tables. Association of hemoglobin levels with intestinal parasite infection was analyzed using logistic regression analyses. Associated background factors including and gestational age, educational status, residence and type of floor was analyzed using binary logistic regression. In binary logistic regression p-value less than 0.05 considered as statistically significant Independent t-test was calculated to find the mean hematological profile difference between parasites infected pregnant women and not-parasite infected pregnant women. Data Quality Assurance

Site assessment and pre-test was done prior to data collection so as to optimize laboratory set up. This was done in samples obtained from 5% of the study participant. All data quality control tools was considered. Data collection was done after the data collectors receive on-site training. Data was checked for completeness prior entry.

Ethical consideration

Ethical clearance was obtained from the research ethical committee of College of Health Science, Debre Markos University. All the information obtained from the study subjects was coded to keep confidential. The study participants were signed written consent.

Result

Socio- demographic characteristics

There were 281 pregnant women who were first trimester participated at the beginning of the study. At second trimester the number of participant decreased to 263 and at third trimester the number of pregnant women were 253. 58.4 %. The study participant's age range were between 26-35 years. Age mean, median and the range of study participant were 26.42, 26, 16 years respectively. Most of the study participants were from urban area. 29.9 % of pregnant women were governmental employer and 44.5% of them were educated college and above (Table 1).

Table1: Socio demographic characteristics of study participate attending antenatal care at Debre Markos Referral hospital from December/ 2017 to February 2019.

character	Frequency	Percentage
**Age category**		
• **18-25**	117	41.6
• **26-35**	164	58.4
Total	281	100
**Residence**		
• Rural	32	11.4

• Urban	248	88.6
Total	281	100
Occupation		
• Government employer	84	29.9
• House wife	72	25.6
• Merchant	64.63	23
• Others	60.37	21.5
Total	281	100
Educational status		
• Not read and write	29	10.3
• Primary	26	9.3
• Secondary	101	35.9
• College and above	125	44.5
Total	281	100

Dietary characteristics and nutritional status of pregnant women

All pregnant women were not taken anti-parasitic drug within the last two weeks before the research has been conducted. But 3.6 % of them were treated with anti -parasitic drug before 1 month of the study began. From the study participant 210 (74.7%) pregnant women were taken iron folate during the first trimester.

Daily food intake and food Varity plays crucial role for pregnant women immunity and hemoglobin level. In this study 220(78.3%) women were took food three times within 24 hours. 79(28.1 %) of pregnant women feed meat, milk or milk product one times within a month. Food intake behaviors of pregnant women varies from person to person. They have good habits of fruit and vegetable intakes. 128(45.6%) pregnant women feed fruit and vegetables at least once within 24 hours (Table 2).

Table 2: Dietary characteristics of pregnant women attending antenatal care at Debre Markos referral hospital, 2019, Ethiopia

Food intake frequency within 24 hours	**Frequency**	**Percent**
• Two times	37	13.2
• Three times	220	78.3
• Four times	24	8.5
Total	281	100
Meat, milk and milk product intake habit		

• Daily	21	7.5
• Two times within week	67	23.8
• One times within week	66	23.5
• One times within month	79	28.1
• One times more than month	48	17.1
**Total**	281	100
Fruit and vegetable intake habit		
• Daily	128	45.6
• Two times within a week	98	34.9
• One times within a week	48	17.1
• One times within a month	1	0.4
• One times more than month	6	2.1
**Total**	281	100

In addition to daily food intake habits of pregnant women; we had assessed Mid- Upper Arm Circumference (MUAC) to estimate nutritional status. Majority of the study participant (79%) MUAC >23 cm. MUAC less than 23 cm considered as an indicator of poor nutritional status. The MUAC mean, media and interquartile range were (25.7 cm, 25 cm and 27.75±7.75 cm)

respectively. In three trimester, the pregnant women nutritional status was almost similar (Table 3)

Table 3.The MUAC feature of pregnant women attending ANC at Debre Markos Referral Hospital at different trimester

Character	Mean	Media	Maximum	Minimum
MUAC				
• First trimester	25.5cm	25cm	33cm	20cm
• Second trimester	25.9cm	25cm	33cm	20cm
• Third trimester	25.7cm	25.5cm	35.5cm	21cm

Prevalence of intestinal parasite among pregnant women

The overall prevalence of intestinal parasite infection among pregnant women was 34.9 %(95% CI: 31.7% -38.2 %). Among these, 281 first trimester pregnant women 94(33.5%) were infected by at list one intestinal parasite. The prevalence of hookworm was 48(17.1 %), *Enteamoeba histolytica* 22(7.8%), *Giardia lamblia* 17(6%) and *Schistosoma mansoni* 9 (3.2 %). Even if, most of the study participant were living in urban area; one third of them were infected by at least one intestinal parasite. The overall prevalence of intestinal parasite among 263 second trimester pregnant women were 33.1 %(95% CI: 27.5- 39.1). Similar with first trimester Hook worm prevalence was highest 29(11%), next *Enteamoeba histolytica* 27(10.7%), *Ascaris lumbricoid*

15(5.7%), *Schistosoma mansoni* 7(2.7), *Giardia lamblia* 5(1.9%), Taenia species 4(1.5%), *Trichuris trichuria* 4 (1.5%) *Strongloid stercolaris* 3(1.1%) and *Hymenolopsis nana* 2(0.8%).

The total prevalence of intestinal parasite among 253 third trimester pregnant women were 97(38.5%) (95% CI: 32.5-44.9). Hookworm consists of the highest prevalence 34(13.4%), *Enteamoeba histolytica* 28(11.1%), *Schistosoma mansonia* 13(5.1%), *Ascaris lumbricoid* 10(4 %), Taenia species10 (4%), *Giardia lamblia* 6(2.4%) *Srongyloid stercolaris* 5(2%), *Hymenolopsis nana* 4(1.6%).

Effects of intestinal parasite on hematological profile

From the study participant 5% (95% CI: 3.5%- 6.8%) of pregnant women were anemic. Their hemoglobin concentration was below 11g/dl. The mean hemoglobin level of intestinal parasite infected and parasite free pregnant women was 12.8 g/dl and 14.4 g/dl respectively. Intestinal parasite infected pregnant women mean HGB, MCV, HCT, MCH and MCHC level were lower than non-infected pregnant women (Table 3).

Table 4: The mean hematological profile of intestinal parasite infected and non-infected pregnant women

Characteristics	Intestinal parasitosis	N	Mean	Std. Deviation
HBG	no	518	14.3643	3.44083
	yes	278	12.8234	1.78701
HCT	no	518	39.7869	4.23747
	yes	278	38.1781	4.39689
MCV	no	518	94.8732	7.23073

	yes	278	94.7464	6.01545
MCH	no	518	33.8728	2.09597
	yes	278	33.1032	3.01313
MCHC	no	518	35.5041	2.16276
	yes	278	34.7061	2.31230

*HGB=Hemoglobin, HCT=Hematocrit, MCV= Mean Corpuscular Volume, MCH= Mean Corpuscular Hemoglobin, MCHC=Mean Corpuscular Hemoglobin Concentration

The Magnitude of mean hemoglobin difference between intestinal parasite free and intestinal parasite infected pregnant women was 1.54g/dl. There was strong association between anemia and intestinal parasite. Those who are infected with hookworm (AOR=21.29, 95%CI 8.28-54.75, *P<0.001*), *Schistosoma mansoni* (AOR=63.73, 95% CI 19.15-212, *p<0.001*) and *Ascaris lumbricoid* (AOR=14.12, 95% CI 3.28-60.65, *P<0.001*) had higher odds of being anemic than non-infected pregnant women (Table 5).

Table 5: The effects of parasite infection on hematological profile of pregnant women

Hematological character	t-test	*p*-value	Mean difference	95% CI
HGB	6.977	<0.001	1.54	1.107-1.974)
HCT	5.04	<0.001	1.61	0.98 -2.21
MCH	4.22	<0.001	0.77	0.41-1.130
MCHC	4.84	<0.001	0.79	0.47-1.120

The prevalence of anemia among first ANC pregnant women was (7.5%). It was strongly associated with intestinal parasitic infection with p<0.001(95 % CI: 2.6 - 21.1). Intestinal parasite

infected pregnant women were 7.47 times more likely develop anemia than non-infected pregnant women. The mean hemoglobin concentration of intestinal parasite infected pregnant women were 12.4g/dl and intestinal parasite free pregnant women were 14.1g/dl. Which indicated parasitic infection has strong effect on hemoglobin concentration of pregnant women.

Anemia prevalence among 2nd trimester pregnant women was 4.9g/dl. Anemia was strongly associated with intestinal parasite infection. Hookworm (p<0.001, AOR=55), *Schistosoma mansoni* (p<0.001, AOR=58), *Ascaris lumbricoid* (P=0.001, AOR=52.8). The mean different hematological profile of intestinal parasite infected pregnant women were HGB=12.8g/dl, HCT=37.9%, MCV =94.9fl, MCH=33.7 pg, MCHC=35.3g/dl. But non-parasitic infected pregnant women were HGB=14.8g/dl, HCT=40.5%, MCV=95.1fl, MCH=33.6 pg, and MCHC=35.5g/dl.

Anemia prevalence among 3rd trimester pregnant women was 2.4 g/dl. Pregnant women at third trimester have better hemoglobin concentration and other hematological profile than first and second trimester pregnant women.

Discussion

There are a number of factors that cause anemia during pregnancy. Among these intestinal parasitic infection is the most common one. In Ethiopia Intestinal parasite is common which have negative effect on mother hemoglobin concentration and infant weight. Therefore, the purpose of this study was to assess the impact of intestinal parasite on hematological profile of pregnant women attending antenatal care at Debre Markos referral hospital.

Few studies are examined to show the burden of intestinal parasite in pregnant women. Intestinal parasite have adverse effect on hematological profile of pregnant women. The mean age of study

participant were 26.42.It is similarly with other study conducted in Ethiopia [9]. To examine the nutritional status of pregnant women we have measured Mid Upper Arm Circumference (MUAC). The mean value of pregnant women MUAC was 27.75±7.75cm. It was higher than other study conducted at Wondo genet district Ethiopia which was 22.8 ±1.9 cm [9]. The difference may be due to population difference, and in our study most of the participant were from urban area which had better life quality.

Pregnant women who had MUAC below 23cm considered as not good nutritional status[10]. As a result in this study pregnant women who had good nutritional status was 79% which was almost similar with other study conducted in south Ethiopia. Women whose MUAC greater than 23cm less likely develop anemia due to nutritional deficiency[11].

Over all prevalence of intestinal parasite among pregnant women attending Antenatal Care (ANC) at Debre Markos referral hospital was 34.9 % (95% CI 31.7% -38.2 %,). Which is in line with other study conducted in Ethiopia with prevalence of 38.7% (95% CI: 33.6–43.8% [12, 13]. But this finding is lower than other study conducted in Indonesia and Venezuela. The prevalence of intestinal parasite among pregnant women was 69.7% and 73.9% respectively [14, 15] and higher than other study conducted in Uganda. The prevalence of intestinal parasite among pregnant women was (16%) [16].The difference might be seasonal variation, population different and study time also one matter. Hook worm prevalence was (11 %) which is higher than other intestinal parasite. Hookworm is one of the most commonly known intestinal parasite that cause anemia. The same study conducted in Ethiopia and Uganda the prevalence of hook worm was similar with our finding [13, 17].

Determining hemoglobin concentration was one of the parameter which was important to assess anemia. In this study the prevalence of anemia among pregnant women was 5% .This finding is lower than other study conducted among Sudan's refuge pregnant women, community based study in western Ethiopia and hospital based study in southern Ethiopia. The prevalence of anemia was 32.2 %, 17.5 % and 31.5% respectively [18-20]. The difference may be due to study area, population and life style that vary from state to state.

In our finding anemia was strongly associated with parasite infection ($p<0.001$). This finding was similar with study conducted in south part of Ethiopia ($p<0.001$).Parasite infected pregnant women was 6.97 more likely develop anemia than parasite free one. The mean hemoglobin difference between parasite not infected and parasite infected was 1.54(95% CI: 1.107-1.974).This finding is in line with similar study conducted in different part of Ethiopia.[9, 11]. Among many factors that cause anemia; intestinal parasite is one factor. In this study hookworm ($p<0.001$), *Schistosoma mansoni* ($p<0.001$) and *Ascaris lumbricoid* ($p<0.001$) were strongly associated with anemia. This finding was similar with other study conducted In Ethiopia[19] and Venzeuela.[2]

The mean hematological profile of pregnant women were (HGB=13.8gm/dl, HCT=39.2%, MCV=94.8fl, MCH=33.6pg, and MCHC=35.2 g/dl). This finding was comparable with the study conducted at Debre Berhan referral hospital of Ethiopia. The hematological profile were HGB=13.8gm/dl, HCT=39.2%, MCV=94.8fl, MCH=33.6pg, and MCHC=35.2 g/dl.[21]. The similarity may be due to age, population and altitude.

The mean hematological difference were observed between parasite infected and parasite free pregnant women. The mean HGB and RBC indices value of intestinal parasite infected were HGB=12.8g/dl, HCT=37.9%, MCV =94.9fl, MCH=33.7pg, MCHC=35.3g/dl. But intestinal

parasite free pregnant women hematological profile were (HGB=14.8g/dl, HCT=40.5%, MCV=95.1fl, MCH=33.6 pg, and MCHC=35.5g/dl).This study is comparable with other study conducted on pregnant women [9, 21].Therefore, parasite infection had adverse impact on hematological profile of pregnant women.

Conclusion

Intestinal parasite infection in pregnant women was common and which had adverse impact on hematological profile. Intestinal parasite infect ted pregnant women hematological profile was decreased which leads anemia in pregnant women as well as on infant and also under birth weight. As to minimize maternal anemia and under birth weight deworming is better before pregnancy.

Acknowledgment

We wish to express our deepest gratitude to Debre Markos University for financial input, materials and other valuable logistic supports that were extremely important for the achievement of this research. Our thanks extend to all members of Debre Markos University, Medical laboratory science staffs, especially there intellectual inputs. Finally we would like to thank for all study participants.

References

1. Obiezue, N.R., et al., *Gastrointestinal Helminth Infection in Pregnancy: Disease Incidence and Hematological Alterations*. Iranian Journal of Public Health, 2013. **42**(5): p. 497-503.
2. Rodriguez-Morales, A.J., et al., *Intestinal parasitic infections among pregnant women in Venezuela*. Infect Dis Obstet Gynecol, 2006. **2006**: p. 23125.
3. Rodríguez-Morales, A.J., et al., *Intestinal Parasitic Infections among Pregnant Women in Venezuela*. Infectious Diseases in Obstetrics and Gynecology, 2006. **2006**: p. 23125.
4. Brooker, S., P.J. Hotez, and D.A. Bundy, *Hookworm-related anaemia among pregnant women: a systematic review*. PLoS Negl Trop Dis, 2008. **2**(9): p. e291.
5. Sousa-Figueiredo, J.C., et al., *Epidemiology of malaria, schistosomiasis, geohelminths, anemia and malnutrition in the context of a demographic surveillance system in northern Angola*. PLoS One, 2012. **7**(4): p. e33189.
6. Mireku, M.O., et al., *Impact of helminth infection during pregnancy on cognitive and motor functions of one-year-old children*. PLoS neglected tropical diseases, 2015. **9**(3): p. e0003463.
7. Williams, J., *District Laboratory Practice in Tropical Countries. Part 1. Monica Cheesbrough. Doddington. Cambridgeshire: Tropical Health Technology, 1998: viii+ 456pp. Price£ 10.90 (surface post) or£ 19.00 (airmail). ISBN 9507434-4-5 [Also available from Cambridge University Press (£ 35.00; ISBN 0-521-66547-7).]*. 2000, Royal Society of Tropical Medicine and Hygiene.
8. Okia, C.C., et al., *Prevalence, Morphological Classification, And Factors Associated With Anemia Among Pregnant Women Accessing Antenatal Clinic At Itojo Hospital, South Western Uganda*. Journal of blood medicine, 2019. **10**: p. 351.

9. Bolka, A. and S. Gebremedhin, *Prevalence of intestinal parasitic infection and its association with anemia among pregnant women in Wondo Genet district, Southern Ethiopia: a cross-sectional study.* BMC Infectious Diseases, 2019. **19**(1): p. 483.
10. Okube, O.T., et al., *Prevalence and factors associated with anaemia among pregnant women attending antenatal clinic in the second and third trimesters at pumwani maternity hospital, Kenya.* Open Journal of Obstetrics and Gynecology, 2016. **6**(1): p. 16-27.
11. Kedir, H., Y. Berhane, and A. Worku, *Khat chewing and restrictive dietary behaviors are associated with anemia among pregnant women in high prevalence rural communities in eastern Ethiopia.* PloS one, 2013. **8**(11): p. e78601.
12. Derso, A., E. Nibret, and A. Munshea, *Prevalence of intestinal parasitic infections and associated risk factors among pregnant women attending antenatal care center at Felege Hiwot Referral Hospital, northwest Ethiopia.* BMC infectious diseases, 2016. **16**(1): p. 530-530.
13. Kefiyalew, F., et al., *Anemia among pregnant women in Southeast Ethiopia: prevalence, severity and associated risk factors.* BMC research notes, 2014. **7**: p. 771-771.
14. Nurdia, D., et al., *Impact of intestinal helminth infection on anemia and iron status during pregnancy: a community based study in Indonesia.* 2001.
15. Rodríguez-Morales, A.J., et al., *Intestinal parasitic infections among pregnant women in Venezuela.* Infectious diseases in obstetrics and gynecology, 2006. **2006**.
16. Ndibazza, J., et al., *Effects of deworming during pregnancy on maternal and perinatal outcomes in Entebbe, Uganda: a randomized controlled trial.* Clinical Infectious Diseases, 2010. **50**(4): p. 531-540.

17. Hailu, T., et al., *Determinant factors of anaemia among pregnant women attending antenatal care clinic in Northwest Ethiopia.* Tropical diseases, travel medicine and vaccines, 2019. **5**: p. 13-13.

18. Alemayehu, A., et al., *Prevalence, Severity, and Determinant Factors of Anemia among Pregnant Women in South Sudanese Refugees, Pugnido, Western Ethiopia.* Anemia, 2016. **2016**: p. 9817358.

19. Mengist, H.M., O. Zewdie, and A. Belew, *Intestinal helminthic infection and anemia among pregnant women attending ante-natal care (ANC) in East Wollega, Oromia, Ethiopia.* BMC Research Notes, 2017. **10**(1): p. 440.

20. Kenea, A., et al., *Magnitude of Anemia and Associated Factors among Pregnant Women Attending Antenatal Care in Public Hospitals of Ilu Abba Bora Zone, South West Ethiopia: A Cross-Sectional Study.* Anemia, 2018. **2018**: p. 9201383.

21. Shitie, D., T. Zewde, and Y. Molla, *Anemia and other hematological profiles of pregnant women attending antenatal care in Debre Berhan Referral Hospital, North Shoa, Ethiopia.* BMC research notes, 2018. **11**(1): p. 704-704.
