# Supplementary material for: Effects of intestinal parasite infection on hematological profiles of pregnant women attending antenatal care at Debre Markos Referral Hospital, Northwest Ethiopia: Institution based prospective cohort study
Source: PLoS One. 2021 May 10;16(5):e0250990. doi: 10.1371/journal.pone.0250990 (PMC8109803; doi:10.1371/journal.pone.0250990)
Supplement: Attachment — Submitted filename: Comments_March_20_2021.pdf [file pone.0250990.s006.pdf]

Effects of intestinal parasite infection on hematological profile of Pregnant Women
Attending Antenatal care in Debre Markos Referral Hospital, North West Ethiopia.
Institution based Prospective cohort study

.

- Data Quality Assurance: It is a subheading but it is not in the proper format.
- It is missing the explanation about how the pregnant women was invited to participate in the study.

Results

- According to the distribution (normal or not) of the quantitative variable it should be indicated **only** the mean or the median and the range or standard deviation of the age.
- It is missing the reasons for the dropout of the participants during each trimester.
- Table 3. It should be better if the p values were shown.

Discussion

- It is missing the discussion about strengths and limitations of the study.

References

- It is not clear the order of the references, please check
