# Supplementary material for: Effects of intestinal parasite infection on hematological profiles of pregnant women attending antenatal care at Debre Markos Referral Hospital, Northwest Ethiopia: Institution based prospective cohort study
Source: PLoS One. 2021 May 10;16(5):e0250990. doi: 10.1371/journal.pone.0250990 (PMC8109803; doi:10.1371/journal.pone.0250990)
Supplement: Attachment — Submitted filename: Comments_Dec_14_2020.docx [file pone.0250990.s003.docx]

**Effects of intestinal parasite infection on hematological profile of Pregnant Women**

**Attending Antenatal care in Debre Markos Referral Hospital, North West Ethiopia.**

**Institution based Prospective cohort study**

**Abstract**

1. Without comments

**Introduction**

- Lines 82-83: The following statement needs a bibliographic support: Many cases of unexplained pregnancy loss are due to undiagnosed tropical diseases.
- In the introduction is missing the aim of the study

**Methods**

- Line 94: Study area, design: both aspects need more details. For example, It should be better if in the study area were indicated the climate and socioeconomic conditions. And the design study needs to be indicated that if it was longitudinal or only a cohort analyzed as a cross sectional with repeated measures.
- Line 97. Study population and sample size determination: It is necessary to add the bibliographic support for the prevalence data used for the sample size calculation
- Lines 130-137. Statistical analysis: It is necessary to indicate if the normal distribution of quantitative variables was assessed.
- Lines 137-138. Data Quality Assurance: It is a subheading but it is not in the proper format.
- Lines 140-141: The following statement needs to be more precise: All data quality control tools was considered.
- It is missing the explanation about how the pregnant women was invited to participate in the study.

**Results**

- Line 152. Age mean, median and the range of study participant were 26.42, 26, 16 years: According to the distribution ( normal or not) of the quantitative variable it should be indicated only the mean or the median and the range or standard deviation of the age.
- It is missing the reasons for the dropout of the participants during each trimester.
- Lines 163-164: This phrase it is proper for the discussion chapter: Daily food intake and food Varity plays crucial role for pregnant women immunity and hemoglobin level.
- Lines 166: the following phrase needs to be more precise: They have good habits of fruit and vegetable intakes.
- Line 178: Table 3. It should be better if the p values were shown.
- Lines 181-196. Prevalence of intestinal parasite among pregnant women: The authors reported the results of stool sample for each trimester but they did not do comparison between them.
- Line 184: This phrase has a spelling mistake: by at *list* one intestinal parasite
- Lines 216-216: this phrase is proper to discussion chapter: Which indicated parasitic infection has strong effect on hemoglobin concentration of pregnant women.

**Discussion**

- Line 233: the following phrase is imprecise: Few studies are examined to show the burden of intestinal parasite in pregnant women.
- Lines 260-261: Taking into account that the main objective of the study was assessed hematological profile, I consider that following phrase needs to be more developed: The prevalence of anemia was 32.2 %, 17.5 % and 31.5% respectively [18-20]. The difference may be due to study area, population and life style that vary from state to state.
- It is missing the discussion about strengths and limitations of the study.
- One part of the conclusions is not corresponding with the results: women as well as on infant and also under birth weight. As to minimize maternal anemia and under birth weight deworming is better before pregnancy.

**References**

- The references number 2,3 and 15 are the same
