# Supplementary material for: Effects of intestinal parasite infection on hematological profiles of pregnant women attending antenatal care at Debre Markos Referral Hospital, Northwest Ethiopia: Institution based prospective cohort study
Source: PLoS One. 2021 May 10;16(5):e0250990. doi: 10.1371/journal.pone.0250990 (PMC8109803; doi:10.1371/journal.pone.0250990)
Supplement: Attachment — Submitted filename: Response to reviewer.docx [file pone.0250990.s005.docx]

PONE-D-20-36685
Effects of intestinal parasite infection on hematological profile of Pregnant Women Attending Antenatal care in Debre Markos Referral Hospital, North West Ethiopia. Institution based Prospective cohort study
PLOS ONE

Response to reviewers

| Question and comments | Response to reviewer |
| --- | --- |
| Reviewer #1: This study is about the effects of intestinal parasite infection on hematological profile of Pregnant Women Attending Antenatal care in Debre Markos Referral Hospital from December 2017 to February 2019. The manuscript needs major revision.   1. In abstract (lines 27-29) and in introduction (lines 90-92); “More than 25% of pregnant women are infected with hookworm, which causes intestinal bleeding and blood loss, and has been most commonly associated with anemia.” According to the reference that the authors refer to it this results is related to Allada in Benin, but the authors wrote it as a general rule, so it has to be revised. 2. .The hematological factors studied in this manuscript give all intestinal parasitic infections the same value. While some of these infections, such as hookworms, may cause more anemia. Therefore, it is necessary to evaluate each parasitic infection separately. 3. The authors evaluated a lot of nutritional factors but the relation between them and parasitic disease is unknown. Are the nutrition status for infected and control group same or not? 4. The pregnant women separated to 3 groups (first, second and third semester) but the results were presented in total.  The control group need to be matched with infected group (Socio- demographic characteristics and dietary characteristics and nutritional status).  The results of Socio- demographic characteristics and dietary characteristics and nutritional status should be expressed in relation to parasitic infection. While in this study these results generally stated. 5. The number of ethical statement needs to add. | 1. We have putted reference 5 and 6 in the introduction part 2. We have corrected all the comments and corrections. I addressed the effects of each intestinal parasite on hematological profile 3. The nutritional status of the study participant was almost similar. Because they are homogenous group. 4. We have wrote the effects of intestinal parasite on hematological profile on 1^st^ ,2^nd^ and 3^rd^ trimester pregnant women separately 5. We have added ethical statement on the paper |
| Reviewer #2 |  |
| Reviewer #2: Reviewer comments Generally the manuscript is important to address effects of IPs among pregnant women; however, it needs major revision. 1. Abstract ¬ Major comments 1. Objective of the study is still a proposal! It does not clearly indicate what objectives were addressed. 2. The method part is not clear!!  3. How many pregnant women were included both in the study and control groups?  4. The data collection (socio-demographic and lab based) should be included!!  How stool and blood samples were collected and processed?  5. What type methods do you used to detect the parasites and anemia parameters?  Method of data analysis is not clearly stated!! At what significant value did you accept the association? It should be included in the abstract data analysis part!!  6. In the result part, the prevalence of IPs should be included since they are the major predisposing factor in pregnant anemia!  7. And also their effect in single and double or triple infections!! Is the anemia mild, moderate or severe both in the infected and control groups? In what percent? 8. The conclusion part should be produced based on your findings!!  9. First what was the prevalence of IPs among pregnant women? Second which parameters of anemia are decreasing due to parasitic infection? Is the anemia mild, moderate or severe?  Poor recommendation? Are anthelminthic drugs safe for pregnant women? I doubt!! Generally the abstract needs major revision!! 10. Minor comments: line no 36, December/ 2017 to February 2019 should be corrected as “December 2017 to February 2019” Revise the grammar!! P-value should be capital on line 48 2. Introduction ¬ Major comments Line no 75-77: in the first place the data is very old and also the reference you put [2] is not a WHO report rather “Rodriguez-Morales, A.J., et al, 2006” data in the reference list. So correction is needed!! 1. Basic information on pregnant associated anemia is not indicated! For instance, 2. What are the possible factors of anemia? It might be caused by physiologically or pathologically during pregnancy. However, you document does not clearly show anemia caused by physiological conditions based on the trimesters. 3. How do you know whether anemia is caused by physiological, malnutrition or parasitic infection??  4. At what parasitic load the parasite can cause anemia (is that in the light, moderate or heavy)? You have to be quite shore to this condition!! Most of the time anemia may be caused by the parasite if and only if the load is high. ⎫ In this study, nutrition and physiological condition of the pregnant are cofounding effects that might lead to false conclusion.  The main objective of the study is not clearly stated in the introduction part!! For instance, 5. Why you want to study in Debre Markos referral hospital? It might be good if you conduct in rural set ups? ⎫ Do you have any institution based information at Debre Markose referral hospital on the level of anemia and prevalence of IPs among pregnant women previously that attract you to conduct this research? ⎫These issues listed above are the major limitation of the introductions parts of your study 6. Minor comments  The grammar should be seen with native speaker  Please do not repeat the whole word and Abbreviations! E.g Soil-transmitted 71 helminths (STHs) line no 71 and 86  3. Methods and materials ¬ Major comments 1. The study area should be full described based on the parameters important for the existence of parasites in the area (altitude, soil-type, Temperature and rainfall. 2. As you mention in the (line no 98-99), you recruited pregnant women who fulfill your inclusion criteria. However, nothing is stated about the inclusion criteria. What was you inclusion criteria? 3. Once the pregnant fulfill the inclusion criteria, how do you select the pregnant women that participated in your study? It is vague whether you select them randomly or conveniently? The sampling frame is generally unclear!! 4. As you mention in the (line no 101-102), to calculate the sample size, you use the prevalence of anemia and among non-helminthic infected (10%) and helminthic infected pregnant women (21.7%), respectively. Where did you get this prevalence? You must indicate the source or reference!!! 5. In the exclusion criteria (line no 105-6), those taking anthelminthic drugs within the last 2 weeks were excluded. In the first place, did pregnant women take anthelminthic drug? At what stage? Secondly, why did you exclude only two weeks? In the re-infection, the parasite cycle needs to be detected by microscopy after 4 weeks. I am not clear about it!! I need your justification or you should revise based on scientific evidences!! 6. How many pregnant women did you select? How did you follow them?  7. When did the lab samples collected? Is that during the 1st, 2nd and 3rd trimester?  8. What did you do for those positive in the 1st trimester? Did you give them treatment and again follow in the 3rd? Completely unclear!!!!! Major limitation! 9. Why did you use direct microscopy and FECT methods together?? FECT might be enough since it is more sensitive than direct saline microscopy!!  The formol ether concentration test (FECT) should be revised critically based on the standard operating procedure!! As far as my knowledge is concerned, half gram of stool is enough to FECT, it is mixed with 10 ml of normal saline and sieved to test tube containing 7 ml of formalin and finally 3 ml of ether is added and the final volume is 10 ml. the revolution per minute is also is not correct!! 10. What parasites did you see with 10x objectives? How did you differentiate the cyst stages of amoeba (you mean by 10X)? Very unusual!!! Totally re-write the FECT principle and procedure clearly!! 11. As you mention in (line no 129), anthropometric measurement was used to assess the nutritional status. What was your guide line and at what point the pregnant women is considered as malnourished? Needs reference!!  12. How did you calculate the prevalence of IP infections and hemoglobin? Descriptive statistic should be included in the analysis part!! 13. Considering the ethical issue, what did you do for those pregnant women infected with IPs and those had low hemoglobin level? Leave the participants untreated is unethical! Generally the method part should be critically modified and needs major revision by considering the study and control groups!!!! ¬ Minor comments: ⎫ line no 93, Methods and materials should be substitute by “Materials and Methods” ⎫ line no 96, December/ 2017 to February 2019 should be corrected as “December 2017 to February 2019” ⎫ line no 105, anti-helminthic should be corrected as “anthelminthic” ⎫ line no 109, Socio demographic should be corrected as “socio-demographic” ⎫ line no 117, 1gm should be corrected as “One gram” ⎫ line no 135, p-value should be corrected as “P-value”  4. Results ¬ Major comments 1. Clearly indicate the socio-demographic characteristics of the study and control groups since it is a cohort study!! 2. Table 1 needs revision! In my knowledge, frequency could not be expressed in ratio rather in number. For instance, merchant and others under the occupation are expressed in ratio.  From line no 163-168, your result showed that the majority of the pregnant women took foods that increase the iron level that also increases the hemoglobin level. My worry is the amount of each food item and the amount iron that they get from each food items is unclear. It is not a matter of eating two, three or four times rather the amount!! This limits your study result!!! 3. In my view, it could have been good if you assess the impacts parasitic infections on hemoglobin level from those pregnant who have MUAC >23 cm since it is possible to remove the cofounding effect of malnutrition (nutrition as a cofounder). What will be your response?? 4. From line 182-195, you reported 33.5%, 33.1% and 38.5% IPs prevalence among pregnant women in the 1st, 2nd and 3rd trimester. I am no clear what do you do during the follow up. Once you get positive in the 1st trimester, you treat them and re-exam in the 2nd and the same thing in the 3rd? Then what did you do for those positive in the 1st trimester? Have treat them and follow them again in the 2nd and 3rd ? This is not clear! You must clearly put the procedure that you follow during the follow up in the method section otherwise; it is very difficult to understand this part of your result.  The prevalence of intestinal parasite among pregnant women both in the control and study groups across their trimesters is not clearly described in the result section. Major limitation!! ⎫ Prevalence of intestinal parasite among pregnant women (both the control and cases) across their trimester might be good if you put in table!  In table 4, it will be very good if you put the normal value in one column so that any reader can easily understand!! 5. From line no 207-210, the odds ratio gap is huge. What could be the possible justification? And also table 5 does not indicate the odds ratio of IPs infections vs anemia! I hope one table is missed that show the association of IPs with hemoglobin!! 6. The severity of anemia (normal, mild, moderate and severe) should be fully indicated both the study and the control groups!!!!! 7. Minor comments ⎫ Line no 151, I am not clear about 253. 58.4%.!! Needs revision specially the percentage!! ⎫ Line no 159 and 160, anti-helminthic should be corrected as “anthelminthic” ⎫ Line no 164, 220(78.3%) should be corrected as “220 (78.3%)”. This should be done throughout the document. ⎫ Line number 167, correct as 128 (45.6%) and numbers could bot ne written in the beginning of a sentences ⎫ Line number 202, (Table 3) should be corrected as (Table 4) ⎫ The grammar should be modified! 5. Discussion ¬ Major comments 1. Line no 228-232, the first paragraph of your discussion is your statement of the problem why you want to conduct this research. It is not placed in its appropriate place. Better to remove it in the discussion part!! 2. In the discussion part, you must discuss your major results in relation to the others study. Needs modification and major revision throughout discussion part!! ¬ Minor comments 3. Line no 252, Hook worm should be corrected as “Hookworm” 4. Line no 262,263,267,268, p<0.001 should be corrected as “P<0.001” 5. . Line no 284, infect ted should be corrected as “infected”  6. Conclusion ⎫ Generally, the conclusion should be drown based on the main findings of your study (pregnant vs parasite, Parasite vs anemia, associated factors with pregnant anemia). 1. The recommendation should be specific to the study paricipants, the community and stakeholders. Generally, these types of studies are important to prevent the burdens of IPs infections among pregnant women in endemic countries since they need strong attention. However, the objectives and the methods of the study should be clearly stated to get a valuable result and to reach in conclusion. It needs major revision and acceptance of it will be secured after the above stated comments are addressed. | 1. We have corrected 2. We have made it clear 3. At the beginning of the study there were 94 intestinal parasite infected and 187 intestinal parasite free pregnant women 4. Stool was collected using leak proof container and venous blood was collected using need with syringe. Wet mount and concentration method was used to detect intestinal parasite and blood was analyzed using hematological analyze (mind ray BC-3000 plus). 5. We have included data analysis method. 6. Corrected as comments 7. Anemia was moderate and mild 8. Corrected based on comments 9. The prevalence of Ip in each trimester was included at the manuscript 10. We have corrected all minor comments.   Introduction   1. We have included few points regarding pregnant associated anemia 2. We have included possible factors that cause anemia 3. To know the cause of anemia among pregnant women we have studied on homogenous groups of pregnant women except intestinal parasite status and statistically we have used-independent t test to analyze the mean hematological profile difference between parasite infected and parasite free pregnant women. So if hematological profile of intestinal parasite pregnant women was decreased. It is due to parasite infection. 4. It is known that parasite cause anemia when parasite load is high. But we have not quantified parasite load. We have used wet mount and formal ether concentration techniques. 5. Actually we did not have published data on intestinal parasite profile at Debre Markos Hospital but we have seen different parasite in the hospital lab when we trained students at Debre Markos referral hospital laboratory. That why we have studied on it to see intestinal parasite effects on hematological profile of pregnant women. 6. All minor comments are corrected   Materia and methods   1. We have included detail about the study area climatic condition and soil characteristics. 2. Those women at first trimester, did not take ant-parasite drug before two weeks, had no chronic disease, considered as study participant. 3. pregnant women was selected randomly 4. sample was calculated using double proportion formula 5. It was editorial problem. Actually pregnant women at first trimester couldn’t take anti parasite drugs. But we have putted it if any special case. 6. The number of pregnant women was 94 and 187 intestinal parasite infected and parasite free pregnant women, respectively. All of pregnant women was at 1^st^ trimester. Blood and stool sample was collected at 1^st^, 2^nd^ and 3^rd^ trimester. 7. Blood and stool sample was collected at 1^st^, 2^nd^ and 3^rd^ trimester. 8. Those who have intestinal parasite at 1^st^ trimester. I have informed for the health care provider in order to follow and I have also informed for the study participant also if there is unusual symptom to communicate with the health care provider. At the 2^nd^ and 3^rd^ trimester I have also informed for the health care provider to treat the study participants. 9. We have used direct microscopy to identify the trophzoite stages of protozoan and we have used concertation method for ova identification 10. We can identify helmithes ova using 10x objectives. 11. MUAC < 23 cm usually considered as indicator of malnutrition. 12. The prevalence of IP and anemia was described using frequency and percentage 13. When the pregnant was positive for IP. At 1^st^ trimester we have informed for health care giver and to follow them and also informed for study participants   at 2^nd^ and 3^rd^ trimester the they were treated by health care givers  Result   1. Accepted and corrected in the manuscript 2. Accepted and corrected 3. There was no statically significant difference between MUAC > 23 cm and MUAC<23 cm. 4. Once if women were positive in the 1st trimester. We have informed for health care provider in order to follow and also informed for the patients, if they had unusual symptoms to contact their clinicians.   In the 2^nd^ trimester if positive treated by health care provider and followed them and again examined in the 3^rd^ trimester, if positive they was treated by the health care provider.   5. The number of infected women was lower. Because of this the odds ratio was huge. 6. Accepted and corrected. 7. All minor comments accepted and corrected   **Discussion**   1. Accepted and removed 2. Accepted and corrected 3. Accepted and corrected 4. Accepted and corrected 5. Accepted and corrected   **Conclusion**   1. Fully accepted the comments and corrected |
| Reviewer #3 |  |
| Reviewer #3: The reviewer of this manuscript would like to appreciate authors for the original work. However, there are issues that require further revision and significant amendment before its further consideration for publication. 1. Topic: Seems good. But there is no/lack of link between the topic and major findings of the study. The topic talks about effect of intestinal parasites on the hematological profile of pregnant women. Whereas, in the result section only data on RBC indices are indicated. While hematological profile refers to all blood cells (including WBC indices) and platelets, which is missing in the document. Rather the study focuses on effect of intestinal parasite on anemia in pregnant women... 2. Abstract, - No justification/rationale, gap to be filled by this work is not indicated - Emphasis has given to data analysis, better to briefly describe the laboratory procedure aspects - Conclusion doesn't supported by the finding, as there is no data on WBCs indices and platelets 3. Introduction, Further refining of available literatures (recent) is important No justification/rationale, gap to be filled by this work is not indicated 4. Method, Sample size is small and difficult to draw conclusion from this, appropriate data collection procedures was used., but data analysis seems shallow and weak to show effect of the infections on RBC indices 5. Discussion (require re-writing) Very shallow and fragmentation/duplication of ideas/contents here and there There is a mix of sections, methods and result parts are in the discussion session (this need further revision and re- writing) Lack of proper scientific reasoning/explanation/justification. e.g. in all cases explanation given to the differences observed in the study from other available literatures are; differences in study area, population and life style of the pregnant women. It could be possible in some case, but can't be in all case. Proper scientific explanation/interpretation is important | 1. Topics   Based on your comments we have added all hematological profiles. Already we had data all hematological profiles but when we analyzed we had leaved the other hematological profile except red blood cell indices and hemoglobin. No, we had included in the manuscript   2. Abstract   -Accepted and corrected   3. Introduction   Tried to included recent documents   4. Methods   Accepted and corrected   5. Accepted and corrected |
| Reviewer #4 |  |
| Reviewer #4: The manuscript has very interesting findings that the authors can analyze and show much better so improve the impact of this publication. Please find in the attachment my comments for each chapter of the manuscript | Accepted and corrected at manuscript |
| Reviewer #5 |  |
| Reviewer #5: Demeke et al. evaluate the impact of intestinal parasite infection on hematological profile of pregnant women attending ANC in Debre Markos Referral Hospital. The study is simple but the subject studied is of public health significance in Ethiopia and could inform policy makers in the efforts to combat intestinal parasitic infection and anemia in pregnancy in the region. While the study seems to have been properly conducted, some issues need to be addressed by the authors.  Major issues  Introduction:  It will benefit readers if the authors could provide a more current WHO statistic on the prevalence and burden of intestinal parasitic infections in Africa and Ethiopia. The introduction also appears to lack information and previous reports on the impact of intestinal parasitic infection on hematological parameters.  Methods:  1. How was the sampling done? Was this random or systematic? A brief description should be provided. 2. The exclusion criteria states that patients with known chronic disease and HIV were not included. Was this information based on word of mouth from the patients or data was retrieved from the hospitals archive. This should be clarified. 3. Age is also a factor that have impact on the hematological profile. Adolescent girls are known to be at high risk for anemia and so are older women. Was there age limit to the study? How many participants were excluded based on the exclusion criteria? A flow chart showing this information may be beneficial to the readers.  4. The authors need to clarify the inclusion/exclusion criteria. Several underling conditions affect the hematological profile.  5. How did the authors ensure that the changes in hematological profile between infected and uninfected participants can be attributed solely to the infection? Line 112: Parasite load could affect whether or not the parasite can be seen in a given sample analyzed using microscopy.  6. Was only a single fecal sample used for the study or multiple sampling per patient?  7. Were the lab analyses done immediately after sample collection?  8. Was there a need for storage?  How was the sample stored? If the sample was also transported, the author should provide a brief description on how this was done. 9. Line 122: Was the slide examination performed by one microscopist? Was only a single slide prepared for each patient? Ideally there should be at least two. The authors should clarify if this was so and also indicate how inconsistent results were resolved. 10. Line 129: Authors should briefly describe which anthropometry was done. Dietary characteristics and nutritional status of pregnant women were also assessed. The authors should describe the tool used. If this was a questionnaire, the authors should indicate whether it was structured/non-structured or whether it was adapted from the WHO guideline. The authors should also be ready and willing to provide a copy of the questionnaire as per the journal’s requirements.  Results:  1. Table 2: It is possible that dietary characteristics of the women may change with time. The total indicates that the nutritional assessment reported is for first trimester. Did the authors assess the same for 2nd and 3rd trimesters? 2.Line 181-96 (Prevalence of intestinal parasite among pregnant women): Only the text has been provided. There is no table/figure to show any of the claims. Line 197-202: The table referencing the effects of intestinal parasite on hematological profile in the text is table 3 (line 202) which rather provides information on MUAC feature of the pregnant women. Table 4 and 5 should be merged. Table 4: The authors appears to have combined 1st, 2nd and 3rd trimester data in this test. This will mean that each patient (minus 1) will have 3 inputs for a given data. The authors should revisit this analysis, separating 1st, 2nd and 3rd trimester data and possibly providing analysis for each. With this, it will be possible to uncover information about which stage of the pregnancy that parasitic infection most relate to anemia in the study region. Line 206-210 appear to talk about the use of logistic regression to identify which parasite has higher odds ratio for anemia but the associated table seem to be a continuation of table 4 (A t test). The authors should check and rectify. Line 212-226: There is no table/figure to support this text.  Discussion:  Line 233: The claim that there are few studies that examine the burden of intestinal parasite in pregnant women is debatable. There are in fact a lot of studies even in Ethiopia alone. The authors should rephrase it. The authors make claims about possible reasons that underlie the differences in findings when compared with other studies (e.g., line 260-61: “The difference may be due to study area, population and life style that vary from state to state.) but fail to provide adequate evidence on what the underlying differences are. How is the study area and population different? What is the lifestyle of the study area that impact the finding but is absent in the study being compared to? A major weakness of the discussion is the lack of synthesis on the public health implications (the “and so what” part of the findings). The discussion and conclusion need to be strengthened. The author claim that the study was a prospective one. It will be of great importance if the author could also provide data on the possible impact of the parasitic infection and the anemia on the birth weight and other clinically relevant data from the babies. The study also has numerous limitations which the authors should indicate. For example, the sample size, although calculated, remains small. Several underlying conditions that could affect the hematological profile were not tested. Although microscopy is useful for detecting parasitic infections, it still presents with substantially low sensitivity that will lead to missed cases especially in the presence of low intensity infections, etc.  Minor issues:  Although the study seems to be thorough, the language is unclear which makes it difficult to follow. The authors, with the help of the journal, could improve the language to make it easier for the readers. The authors should also avoid repeating the results (numbers and percentages) in the discussion. Paraphrasing the main findings will be beneficial. The authors may also want to also revisit the referencing of texts. Some statements are made (such as line 623) without a reference. The authors may want to avoid starting sentences with numbers. There is also no need to be stating the total of each variable in table 1 if all sum up to the overall total. “Not read and write” can also be changed to “No formal education”. Line 102-3 “Pregnant women who attend antenatal care (ANC) unit at Debre Markos referral hospital during the study period” appears incomplete. | Method   1. Consecutive convenience 2. Data was retrieved from hospital 3. There was no age limitation but we had tried to see effects of age on hematological profile but had no significant difference. 4. Accepted and corrected 5. By comparing the mean difference between infected and parasite free pregnant women. We had also assessed nutritional status to avoid confounding factors. 6. Three stool samples was collected from each patients in every trimester 7. Sample was examined within 15 minutes after collection for trophozoite identification and the remaining sample was preserved using 10% formalin 8. Preserved and transported to Debre markos university for formal-ethor concentration techniques 9. The examination was performed by three Medical parasitologist 10. Accepted   **Result**   1. The nutritional status of pregnant women was only assessed at the beginning of the study at 1^st^ trimester. 2. Accepted and corrected |
| Reviewer #6 |  |
| Reviewer #6: Dear PloS One editor, Here are the general comments for the author. The comments are briefly described in the main manuscript and high-lighted using yellow texts and accessible to the author in charge of the manuscript for reaction. To highlight some of the comments, Abstract To some extent, it doesn’t reflect the main manuscript, especially of methods and result part. So, it needs re-construction. Methods 1. Study design: requires justification for study design to be cohort and prospective. The result gives some clues for it to be more likely cross-sectional type and retrospective despite of the long data collection period 2. The sample size is not known and it lacks calculation for computing sample size 3. The sampling technique is not stated 4. The issue of ethical approval needs evidence like approval number should be stated or approval letter should be attached/submitted Result 5. In general, the organization is poor; statistical tools are not well described especially the write-up for example while writing OR with 95% C.I and P-value o There is not table that describes multivariate analysis o Table 2&3, their importance is under question in line with the study objective o There are many grammatical and spelling errors in result description o In table 5, it lacks descriptions concerning t-test result like for example the finding and its relevance Discussion o In summary, it lacks organized way of write-up and coherence. I kindly recommend improvement including grammatical and spelling errors corrections by using online grammar checker Conclusion o Some of it is not based on the result output. So, kindly correct it References o Citations are not according their order in the reference section | 1. At the beginning of the study, the study participants were 281 pregnant women who were at first trimester. Among these pregnant women 94 were intestinal parasite infected and 187 were free from parasite. At the second trimester eighteen of them were absent from follow up. At the third trimester 10 of them were absent. Only 253 were followed till the end of the study 2. The sample size was calculated using double population proportion formal. It was clearly describe at the manuscript methodology part 3. Random sampling 4. We have included approval number   All are corrected accordingly |
| Reviewer #7 |  |
| Reviewer #7: Although the authors addressed an interesting topic, the manuscript is very preliminary. In addition, there are problems on results presentation and and writing. Besides that the results and the conclusion cannot be supported by the results presented. | We have corrected all comments and suggestions |
|  |  |
