# Supplementary material for: Effects of intestinal parasite infection on hematological profiles of pregnant women attending antenatal care at Debre Markos Referral Hospital, Northwest Ethiopia: Institution based prospective cohort study
Source: PLoS One. 2021 May 10;16(5):e0250990. doi: 10.1371/journal.pone.0250990 (PMC8109803; doi:10.1371/journal.pone.0250990)
Supplement: Attachment — Submitted filename: Response to reviewers.doc [file pone.0250990.s007.doc]

Response to Reviewers

Dear Editor,

PLOS ONE

First, we would like to give our great thanks and appreciation to editor and reviewers for their critical review of our paper entitled: “**Effects of intestinal parasite infection on hematological profile of pregnant women attending antenatal care in Debre Markos Referral Hospital, North West Ethiopia: Institution based prospective cohort study”**

By: Gebreselassie Demeke ^1*,^ Getachew Mengistu ^2^, Abtie Abebaw^3^_,_ Milkiyas Toru ^4^, Molla Yigzaw ^5,^ Aster Shiferaw^6^, Hylemariam Mihiretie^7^, Tebelay Dilnessa (MSc.) ^8^

1. Department of Medical Laboratory Science, College Health Science, Debre Markos University, Debre Markos, Ethiopia; e-mail: gebredemeke@yahoo.com
2. Department of Medical Laboratory Science, College Health Science, Debre Markos University, Debre Markos, Ethiopia; e-mail: [gechomlt@gmail.com](mailto:gechomlt@gmail.com)
3. Department of Medical Laboratory Science, College Health Science, Debre Markos University, Debre Markos, Ethiopia; e-mail: [aabtie@gmail.com](mailto:aabtie@gmail.com)
4. Department of Medical Laboratory Science, College Health Science, Debre Markos University, Debre Markos, Ethiopia; e-mail: [milkiastoru@gmail.com](mailto:milkiastoru@gmail.com)
5. Department of Medical Laboratory Science, College Health Science, Debre Markos University, Debre Markos, Ethiopia; e-mail: [mollayigzaw33@gmail.com](mailto:mollayigzaw33@gmail.com)
6. Department of Midwifery, College Health Science, Debre Markos University, Debre Markos, Ethiopia; e-mail: [astershiferaw21@gmail.com](mailto:astershiferaw21@gmail.com)
7. Department of Medical Laboratory Science, College Health Science, Debre Markos University, Debre Markos, Ethiopia; e-mail: hylemariam@gmail.com
8. Department of Medical Laboratory Science, College Health Science, Debre Markos University, Debre Markos, Ethiopia; e-mail: tebelay@gmail.com

*Correspondence Author:

Gebreselassie Demeke, email address: [gebredemeke@yahoo.com](mailto:gebredemeke@yahoo.com), telephone: +251924338828

Dear Editor,

First, we would to acknowledge you for the comment forwarded which is very important for the improvement of the paper. We tried to give a point by point response to each comment. All comments were accepted and incorporated to the manuscript. We also made a proofreading through the document. Reference citations were also updated and corrected with respect to the modification made.

Ethical Corrections

1. **Editor**: Please review your reference list to ensure that it is complete and correct. If you have cited papers that have been retracted, please include the rationale for doing so in the manuscript text, or remove these references and replace them with relevant current references. Any changes to the reference list should be mentioned in the rebuttal letter that accompanies your revised manuscript. If you need to cite a retracted article, indicate the article’s retracted status in the References list and also include a citation and full reference for the retraction notice.

**Author response**: We have checked all references which are re-organized and correctly cited and we also added one additional reference which supports the already existing documents. Reference number 2

**Response to reviewer**

1. **Reviewer #1:** In this study the authors evaluated the effects of intestinal parasite infection on hematological profile of Pregnant Women.
   It seems that the authors revised the manuscript according to the comments.

**Author response**: Thank you for your critical comments to our paper. We accepted all comments and incorporated to the paper.

1. **Reviewer # 2**:  Authors have addressed many of my comments which was given previously but still the grammar part is a critical problem throughout the document. So, much work should be done on the grammar issues.

**Author response**: We have accepted all comments and corrections. We did a proofreading through the document. As much as possible we have tried to correct all grammatical issues.

2. **Reviewer # 4**: Thank you for taking into account the most of my comments and for your hard work. However, it still is necessary to address some few comments. Please check in the attachment file

**Materials and Methods**

3. **Reviewer # 4:** Data Quality Assurance: It is a subheading but it is not in the proper format.

**Author response**: Accepted and corrected on the manuscript

4. **Reviewer # 4:** It is missing the explanation about how the pregnant women was invited
   to participate in the study

**Author response:** Those pregnant women who came at Debre Markos Referral Hospital follow antenatal care were invited to participate in the study. From those who fulfil inclusion criteria and voluntary to sign consent were considered as study participants. From voluntary pregnant women the study participants were selected conveniently.

**Result section**

5. **Reviewer # 4** According to the distribution (normal or not) of the quantitative
   variable it should be indicated **only** the mean or the median and the
   range or standard deviation of the age.

**Author response**: We have accepted and corrected at manuscript. We used mean and standard deviation.

6. Reviewer # 4: It is missing the reasons for the dropout of the participants during each
   trimester.

**Author response**: Based on your comments we have included the reason for participants dropout. The study participates absenteeism was due to facility change, transfer to nearby facility, absent from follow up due to unknown reason and some of them were refused to give biological sample especially at third trimester.

**Discussion section**

7. **Reviewer # 4 :** It is missing the discussion about strengths and limitations of the study

**Author response:** we have accepted the comments and we included strength and limitation of our study.

**Reference**

8. **Reviewer # 4 :** It is not clear the order of the references, please check

**Author response:** accepted and checked

9. **Reviewer # 6** Despite addressing majority of the comments, still a few comments to the author:
   1. The tables in general are poorly organized. So. I kindly ask the author to organize the lay out of the tables so that it can fit to one page preferably. Minimize the space between the rows.

**Author response:** We have accepted the comments. We tried to reorganized the table and the space between raw is minimized.

10. **Reviewer # 6:** There noticed grammatical error for example in line 199. It says " didn't taken

**Author response:** We did proof reading throughout the document and as much as possible the grammatical errors are corrected

Thank you for all Editors and Reviewers.
