# Supplementary material for: Effects of intestinal parasite infection on hematological profiles of pregnant women attending antenatal care at Debre Markos Referral Hospital, Northwest Ethiopia: Institution based prospective cohort study
Source: PLoS One. 2021 May 10;16(5):e0250990. doi: 10.1371/journal.pone.0250990 (PMC8109803; doi:10.1371/journal.pone.0250990)
Supplement: Attachment — Submitted filename: Response to reviewers.doc [file pone.0250990.s008.doc]

Dear Editor,

PLOS ONE

First, we would like to give our great thanks and appreciation to editor and reviewers for their critical review of our paper entitled: “**Effects of intestinal parasite infection on hematological profiles of pregnant women attending antenatal care in Debre Markos Referral Hospital, northwest Ethiopia: Institution based prospective cohort study” to be published at PLOS ONE.**

By: Gebreselassie Demeke ^1*,^ Getachew Mengistu ^2^, Abtie Abebaw^3^_,_ Milkiyas Toru ^4^, Molla Yigzaw ^5,^ Aster Shiferaw^6^, Hylemariam Mihiretie Mengist^7^, Tebelay Dilnessa ^8^

1. Department of Medical Laboratory Science, College Health Science, Debre Markos University, Debre Markos, Ethiopia; e-mail: gebredemeke@yahoo.com
2. Department of Medical Laboratory Science, College Health Science, Debre Markos University, Debre Markos, Ethiopia; e-mail: [gechomlt@gmail.com](mailto:gechomlt@gmail.com)
3. Department of Medical Laboratory Science, College Health Science, Debre Markos University, Debre Markos, Ethiopia; e-mail: [aabtie@gmail.com](mailto:aabtie@gmail.com)
4. Department of Medical Laboratory Science, College Health Science, Debre Markos University, Debre Markos, Ethiopia; e-mail: [milkiastoru@gmail.com](mailto:milkiastoru@gmail.com)
5. Department of Medical Laboratory Science, College Health Science, Debre Markos University, Debre Markos, Ethiopia; e-mail: [mollayigzaw33@gmail.com](mailto:mollayigzaw33@gmail.com)
6. Department of Midwifery, College Health Science, Debre Markos University, Debre Markos, Ethiopia; e-mail: [astershiferaw21@gmail.com](mailto:astershiferaw21@gmail.com)
7. Department of Medical Laboratory Science, College Health Science, Debre Markos University, Debre Markos, Ethiopia; e-mail: hylemariam@gmail.com
8. Department of Medical Laboratory Science, College Health Science, Debre Markos University, Debre Markos, Ethiopia; e-mail: tebelay@gmail.com

*Correspondence Author:

Gebreselassie Demeke, email address: [gebredemeke@yahoo.com](mailto:gebredemeke@yahoo.com), telephone: +251924338828

Dear Editor,

First, we would like to acknowledge you for the comment forwarded which is very important for the improvement of the paper. All comments were accepted and incorporated to the manuscript. We also made a proofreading through the document.

1. **Editor**:  It seems as if the authors have responded to most of the reviewers' comments. However, an issue still stands with English grammar and syntax. For instance, there are words than need hyphenating, like "intestinal parasite-free pregnancies" and there are several incomplete sentences, among other issues. You must contact a copyeditor/colleague that has expertise in the area of English writing and composition before resubmitting this manuscript.
2. **Author response**: All English grammar, syntax, incomplete sentences, and hyphenating are corrected by experts.
