# Supplementary material for: Effects of intestinal parasite infection on hematological profiles of pregnant women attending antenatal care at Debre Markos Referral Hospital, Northwest Ethiopia: Institution based prospective cohort study
Source: PLoS One. 2021 May 10;16(5):e0250990. doi: 10.1371/journal.pone.0250990 (PMC8109803; doi:10.1371/journal.pone.0250990)
Supplement: Attachment — Submitted filename: Reviewer comments to authors.pdf [file pone.0250990.s002.pdf]

Reviewer comments

Generally the manuscript is important to address effects of IPs among pregnant women; however, it needs major revision.

1. **Abstract**
   - **Major comments**
     - ✓ Objective of the study is still a proposal! It does not clearly indicate what objectives were addressed.
     - ✓ The method part is not clear!!
     - ✓ How many pregnant women were included both in the study and control groups?
     - ✓ The data collection (socio-demographic and lab based) should be included!!
     - ✓ How stool and blood samples were collected and processed?
     - ✓ What type methods do you used to detect the parasites and anemia parameters?
     - ✓ Method of data analysis is not clearly stated!! At what significant value did you accept the association? It should be included in the abstract data analysis part!!
     - ✓ In the result part, the prevalence of IPs should be included since they are the major predisposing factor in pregnant anemia! And also their effect in single and double or triple infections!! Is the anemia mild, moderate or severe both in the infected and control groups? In what percent?
     - ✓ The conclusion part should be produced based on your findings!! First what was the prevalence of IPs among pregnant women? Second which parameters of anemia are decreasing due to parasitic infection? Is the anemia mild, moderate or severe?
     - ✓ Poor recommendation? Are anthelminthic drugs safe for pregnant women? I doubt!! Generally the abstract needs major revision!!
   - **Minor comments:**
     - ✓ line no 36, December/ 2017 to February 2019 should be corrected as "December 2017 to February 2019"
     - ✓ Revise the grammar!! P-value should be capital on line 48
2. **Introduction**
   - **Major comments**
     - ✓ Line no 75-77: in the first place the data is very old and also the reference you put [2] is not a WHO report rather "Rodriguez-Morales, A.J., et al, 2006" data in the reference list. So correction is needed!!
     - ✓ Basic information on pregnant associated anemia is not indicated! For instance,
     - ✓ What are the possible factors of anemia? It might be caused by physiologically or pathologically during pregnancy. However, you document does not clearly show anemia caused by physiological conditions based on the trimesters.
     - ✓ How do you know whether anemia is caused by physiological, malnutrition or parasitic infection??

- ✓ At what parasitic load the parasite can cause anemia (is that in the light, moderate or heavy)? You have to be quite shore to this condition!! Most of the time anemia may be caused by the parasite if and only if the load is high.
- ✓ In this study, nutrition and physiological condition of the pregnant are cofounding effects that might lead to false conclusion.
- ✓ The main objective of the study is not clearly stated in the introduction part!! For instance,
- ✓ Why you want to study in Debre Markos referral hospital? It might be good if you conduct in rural set ups?
- ✓ Do you have any institution based information at Debre Markose referral hospital on the level of anemia and prevalence of IPs among pregnant women previously that attract you to conduct this research?
- ✓ These issues listed above are the major limitation of the introductions parts of your study

➢ **Minor comments**

- ✓ The grammar should be seen with native speaker
- ✓ Please do not repeat the whole word and Abbreviations! E.g Soil-transmitted 71 helminths (STHs) line no 71 and 86

3. Methods and materials

➢ **Major comments**

- ✓ The study area should be full described based on the parameters important for the existence of parasites in the area (altitude, soil-type, Temperature and rainfall.
- ✓ As you mention in the (line no 98-99), you recruited pregnant women who fulfill your inclusion criteria. However, nothing is stated about the inclusion criteria. What was you inclusion criteria?
- ✓ Once the pregnant fulfill the inclusion criteria, how do you select the pregnant women that participated in your study? It is vague whether you select them randomly or conveniently? The sampling frame is generally unclear!!
- ✓ As you mention in the (line no 101-102), to calculate the sample size, you use the prevalence of anemia and among non-helminthic infected (10%) and helminthic infected pregnant women (21.7%), respectively. Where did you get this prevalence? You must indicate the source or reference!!!
- ✓ In the exclusion criteria (line no 105-6), those taking anthelminthic drugs within the last 2 weeks were excluded. In the first place, did pregnant women take anthelminthic drug? At what stage? Secondly, why did you exclude only two weeks? In the re-infection, the parasite cycle needs to be detected by microscopy after 4 weeks. I am not clear about it!! I need your justification or you should revise based on scientific evidences!!
- ✓ How many pregnant women did you select? How did you follow them? When did the lab samples collected? Is that during the 1st, 2nd and 3rd trimester? What did you do for those positive in the 1st trimester? Did you give them treatment and again follow in the 3rd? Completely unclear!!!!! Major limitation!

- ✓ Why did you use direct microscopy and FECT methods together?? FECT might be enough since it is more sensitive than direct saline microscopy!!
- ✓ The formol ether concentration test (FECT) should be revised critically based on the standard operating procedure!! As far as my knowledge is concerned, half gram of stool is enough to FECT, it is mixed with 10 ml of normal saline and sieved to test tube containing 7 ml of formalin and finally 3 ml of ether is added and the final volume is 10 ml. the revolution per minute is also is not correct!!
- ✓ What parasites did you see with 10x objectives? How did you differentiate the cyst stages of amoeba (you mean by 10X)? Very unusual!!! Totally re-write the FECT principle and procedure clearly!!
- ✓ As you mention in (line no 129), anthropometric measurement was used to assess the nutritional status. What was your guide line and at what point the pregnant women is considered as malnourished? Needs reference!!
- ✓ How did you calculate the prevalence of IP infections and hemoglobin? Descriptive statistic should be included in the analysis part!!
- ✓ Considering the ethical issue, what did you do for those pregnant women infected with IPs and those had low hemoglobin level? Leave the participants untreated is unethical!
- ✓ Generally the method part should be critically modified and needs major revision by considering the study and control groups!!!!

➢ **Minor comments:**

- ✓ line no 93, Methods and materials should be substitute by “Materials and Methods”
- ✓ line no 96, December/ 2017 to February 2019 should be corrected as “December 2017 to February 2019”
- ✓ line no 105, anti-helminthic should be corrected as “anthelminthic”
- ✓ line no 109, Socio demographic should be corrected as “socio-demographic”
- ✓ line no 117, 1gm should be corrected as “One gram”
- ✓ line no 135, p-value should be corrected as “*P-value*”

4. Results

➢ **Major comments**

- ✓ Clearly indicate the socio-demographic characteristics of the study and control groups since it is a cohort study!!
- ✓ Table 1 needs revision! In my knowledge, frequency could not be expressed in ratio rather in number. For instance, merchant and others under the occupation are expressed in ratio.
- ✓ From line no 163-168, your result showed that the majority of the pregnant women took foods that increase the iron level that also increases the hemoglobin level. My worry is the amount of each food item and the amount iron that they get from each food items is unclear. It is not a matter of eating two, three or four times rather the amount!! This limits your study result!!!
- ✓ In my view, it could have been good if you assess the impacts parasitic infections on hemoglobin level from those pregnant who have MUAC >23 cm since it is possible to

remove the cofounding effect of malnutrition (nutrition as a cofounder). What will be your response??

- ✓ From line 182-195, you reported 33.5%, 33.1% and 38.5% IPs prevalence among pregnant women in the 1st, 2nd and 3rd trimester. I am no clear what do you do during the follow up. Once you get positive in the 1st trimester, you treat them and re-exam in the 2nd and the same thing in the 3rd? Then what did you do for those positive in the 1st trimester? Have treat them and follow them again in the 2nd and 3rd ? This is not clear! You must clearly put the procedure that you follow during the follow up in the method section otherwise; it is very difficult to understand this part of your result.
- ✓ The prevalence of intestinal parasite among pregnant women both in the control and study groups across their trimesters is not clearly described in the result section. Major limitation!!
- ✓ Prevalence of intestinal parasite among pregnant women (both the control and cases) across their trimester might be good if you put in table!
- ✓ In table 4, it will be very good if you put the normal value in one column so that any reader can easily understand!!
- ✓ From line no 207-210, the odds ratio gap is huge. What could be the possible justification? And also table 5 does not indicate the odds ratio of IPs infections vs anemia! I hope one table is missed that show the association of IPs with hemoglobin!!
- ✓ The severity of anemia (normal, mild, moderate and severe) should be fully indicated both the study and the control groups!!!!!

- ➢ **Minor comments**
  - ✓ Line no 151, I am not clear about 253. 58.4%.!! Needs revision specially the percentage!!
  - ✓ Line no 159 and 160, anti-helminthic should be corrected as “anthelminthic”
  - ✓ Line no 164, 220(78.3%) should be corrected as “220 (78.3%)”. This should be done throughout the document.
  - ✓ Line number 167, correct as 128 (45.6%) and numbers could bot ne written in the beginning of a sentences
  - ✓ Line number 202, (Table 3) should be corrected as (Table 4)
  - ✓ The grammar should be modified!

5. Discussion

- ➢ **Major comments**
  - ✓ Line no 228-232, the first paragraph of your discussion is your statement of the problem why you want to conduct this research. It is not placed in its appropriate place. Better to remove it in the discussion part!!
  - ✓ In the discussion part, you must discuss your major results in relation to the others study. Needs modification and major revision throughout discussion part!!
- ➢ **Minor comments**
  - ✓ Line no 252, Hook worm should be corrected as “Hookworm”
  - ✓ Line no 262,263,267,268, p<0.001 should be corrected as “*P<0.001*”
  - ✓ Line no 284, infect ted should be corrected as “infected”

6. Conclusion

- ✓ Generally, the conclusion should be drown based on the main findings of your study (pregnant vs parasite, Parasite vs anemia, associated factors with pregnant anemia).
- ✓ The recommendation should be specific to the study paricipants, the community and stakeholders.

Generally, these types of studies are important to prevent the burdens of IPs infections among pregnant women in endemic countries since they need strong attention. However, the objectives and the methods of the study should be clearly stated to get a valuable result and to reach in conclusion. It needs major revision and acceptance of it will be secured after the above stated comments are addressed.
